# Reply to Giles N. Cattermole and Mike Wells

**DOI:** 10.29045/14784726.2021.6.6.1.54

**Published:** 2021-05-01

**Authors:** Karl Charlton, Matt Capsey, Chris Moat

**Affiliations:** North East Ambulance Service NHS Foundation Trust; Teesside University; Teesside University

Thank you to Cattermole and Wells for your response to our publication and we welcome the discussion related to our findings. It is pleasing to note that the respondents agree with our findings that estimation of weight based on age is inaccurate and risks mis-dosing of some drugs. The overall aim of our research was to analyse the accuracy of paediatric weight estimation by UK ambulance personnel applying age-based methods of estimating weight using contemporary data. The purpose of our paper was not to compare actual weight to parental estimation or any other tools, as this information was not available in our dataset. Our intention was to critique/analyse current weight estimation tools but not to undermine existing practitioners.

In the implications for practice section, we stated that if an accurate weight is available, it should be the basis for practice. We were also mindful of potential readers in clinical practice who would like to know what they should do with the resources currently available. Page for age is reliable for those drugs that are dosed for age; where no superior estimation method is available (e.g. scales, known weight or tape-based tools), then clinicians should continue to follow the page for age.

It was our intention to continue the discussion for improving clinical practice and we are pleased to see this dialogue developing. We are hoping to continue this area of research, and one area to be considered is the analysis of other methods of paediatric weight estimation.

